# Artificial Intelligence for Mechanical Ventilation: A Transformative Shift in Critical Care

**DOI:** 10.1177/29768675241298918

**Published:** 2024-11-11

**Authors:** Giovanni Misseri, Matteo Piattoli, Giuseppe Cuttone, Cesare Gregoretti, Elena Giovanna Bignami

**Affiliations:** Anaesthesiology and Intensive Care Unit, 97860 Fondazione Istituto “G. Giglio”, Cefalù, Palermo, Italy; Saint Camillus International University of Health and Medical Sciences “UniCamillus”, Rome, Italy; 9311 Università degli Studi di Roma “La Sapienza”, Rome, Italy; 217140 Università degli Studi di Enna “Kore”, Enna, Italy; Anaesthesiology and Intensive Care Unit, 97860 Fondazione Istituto “G. Giglio”, Cefalù, Palermo, Italy; Saint Camillus International University of Health and Medical Sciences “UniCamillus”, Rome, Italy; Department of Medicine and Surgery, Anaesthesiology, Critical Care and Pain Medicine Division, Azienda Ospedaliero-Universitaria di Parma, 9370 Università di Parma, Parma, Italy

**Keywords:** artificial intelligence, AI, critical care, intensive care, machine learning, mechanical ventilation, personalized medicine

## Abstract

With the large volume of data coming from implemented technologies and monitoring systems, intensive care units (ICUs) represent a key area for artificial intelligence (AI) application. Despite the last decade has been marked by studies focused on the use of AI in medicine, its application in mechanical ventilation management is still limited. Optimizing mechanical ventilation is a complex and high-stake intervention, which requires a deep understanding of respiratory pathophysiology. Therefore, this complex task might be supported by AI and machine learning. Most of the studies already published involve the use of AI to predict outcomes for mechanically ventilated patients, including the need for intubation, the respiratory complications, and the weaning readiness and success. In conclusion, the application of AI for the management of mechanical ventilation is still at an early stage and requires a cautious and much less enthusiastic approach. Future research should be focused on AI progressive introduction in the everyday management of mechanically ventilated patients, with the aim to explore the great potentiality of this tool.

## Plain Language Summary

The use of artificial intelligence for mechanical ventilation

Artificial Intelligence (AI) could help the management of patients treated with mechanical ventilation in critical care practice. Current guidelines are based on data coming from the general population, without considering the individual patients' characteristics. With the use of AI for mechanical ventilation, critical care practice could be improved by offering personalized treatments, reducing complications, and assisting clinicians in decision-making to improve patient outcomes and reduce mortality rates. Despite AI in medicine has progressed in the last decade, little is known about its use in critical care and in ventilation management. In order to improve its everyday use, future research should be performed in intensive care settings.

## Introduction

With the large volume of data coming from implemented technologies and monitoring systems, intensive care units (ICUs) represent a key area for leveraging artificial intelligence (AI) to enhance patient care and outcomes through personalization and optimization of clinical decisions.^1^

It is in this perspective that management of mechanical ventilation might benefit from integration of AI in critical care practice. Optimizing mechanical ventilation is a complex and high-stake intervention, requiring precise and continuous adjustments. This task is further complicated by the heterogeneity of patients’ responses, due to the variability in the underlying causes of the respiratory conditions being treated, lung mechanics and individual physiological characteristics.^2^ Current guidelines and best practices on mechanical ventilation are based on results coming from clinical trials conducted at a population level. This often leads to not meeting the individual patient's needs, and to an inappropriate clinical management that may potentially increase morbidity and mortality.

As a result, the application of AI in mechanical ventilation might represent a transformative shift in critical care, offering a personalized approach while reducing complications,^3^ potentially improving outcomes, and assisting intensivists in their clinical decisions.

## Applications of AI in ICU

Early disease identification, prediction of patients’ clinical evolution, personalized treatment strategies and optimization of healthcare resources allocation are to be considered the future promises of AI application in critical care.^4^

Despite the use of AI in ICU is still taking its first steps, several studies have so far revealed the potentials of this technology in the management of critically ill patients. Some of these used big data sets in order to predict length of stay and mortality,^5^ while others applied AI for early detection of sepsis and septic shock,^6,7^ cardiocirculatory failure,^8^ and acute respiratory conditions.^2^

In their recent validation study, Persson et al^9^ tested the NAVOY sepsis algorithm demonstrating its ability to detect patients at high risk to develop sepsis within 3 h. This algorithm revealed a prediction performance superior to existing sepsis early warning scoring systems (eg, SOFA, qSOFA, MEWS, NEWS2), showing its usefulness if integrated into routine clinical practice.

The Feasible Artificial Intelligence with Simple Trajectories for Predicting Adverse Catastrophic Events model can predict the onset of cardiac arrest or acute respiratory failure from 1 h to 6 h prior to its occurrence (AUROC 0.886 and 0.869, for the 2 respective outcomes).^10^

As previously outlined, the use of AI in ICU environments is mainly limited to machine learning which combines statistical analysis techniques with computer science to produce algorithms aimed at generating knowledge from available data,^5^ but with no actual intervention on events. Even if this application of AI technology would be of great assistance for intensivists dealing with information overload and the need to make quick decisions, the “predictive” AI approach should be complemented by an “actionable” AI approach.^11^ This refers to casual inference, or the ability to predict outcomes and events that would result from alternative decisions/treatments. Hence, the comparison of different future potential outcomes deriving from different decisions/treatments should lead AI to identify “the best possible predicted outcome,” and therefore choose the optimal decision/treatment.

## The Use of AI for Mechanical Ventilation Management

More than any other device in ICU, mechanical ventilators offer a large amount of data as settings, waveforms, alarms, and measured parameters.^12^ When integrated with clinical variables and patient characteristics, it is reasonable to expect that the implementation of AI might improve efficiency, efficacy, and safety in critical care.

According to a recent systematic review,^2^ most studies involve the use of AI to predict outcomes for mechanically ventilated patients, including the need for mechanical ventilation, the complications, and the weaning success.

Timely identification of patients developing ARDS and risk stratification through AI implementation has been explored in different studies.^12–14^ Interestingly, by combining structured (monitor and laboratory data) and unstructured data (clinical notes), Apostolova et al^14^ applied a deep learning approach to build context vectors containing information on patients’ conditions, which were then combined together and analyzed by a prediction model in order to successfully identify early development of ARDS.

Another potential advantage of AI implementation for mechanical ventilation practice is its ability to identify specific phenotypes and personalize treatments accordingly: hypo- and hyperinflammatory ARDS phenotypes might in fact benefit from different therapeutic approaches.^15^ With regard to ventilation, AI may indicate the most correct strategy which may be beneficial for the considered ARDS subphenotype^15^ in *real time* and modify ventilator parameters accordingly.

Mechanical ventilation is admittedly an “open-loop” system, where the input (the set ventilation mode) is not influenced by the output (the adequacy of the ventilation settings)^5^: an ideal model should adjust the ventilator settings while analyzing respiratory mechanics and considering potential clinical improvements. Thus, “closed-loop” newer ventilation modes could target complex purposes such as prevention of ventilator-induced lung injury, continuously adapting to lung mechanics and patient conditions, while even testing weaning success and extubation readiness. In this respect, it should be noted that the currently commercially available mode INTELLiVENT–Adaptive Support Ventilation (INTELLiVENT–ASV^®^, Hamilton Medical) has thus far proven to be clinically safe and to effectively reduce healthcare team workload by reducing manual setting adjustments.^16^

Patient-ventilator asynchronies too have been extensively explored, given how lack of adequate patient-ventilator coupling is known to be associated with higher mortality and delayed extubation. Sottile et al^17^ applied a number of machine learning algorithms on data from 62 ventilated patients at risk for, or affected by ARDS. In their study, they were able to identify synchronous breathing and presence of asynchronies (double triggering, flow limitation, and ineffective triggering) with high sensitivity and specificity. In their pilot study, Gholami et al^18^ used a machine learning framework to automatically and continuously detect cycling asynchronies based on waveform analysis: this model detected the presence of cycling asynchronies with a sensitivity and specificity of 89% and 99%, respectively. The results of these and other studies may represent a turning point in mechanical ventilation, enabling clinicians to adequately respond to alerts while ameliorating ventilation management.

The VentAI^19^ is a reinforcement learning algorithm which is able to suggest a dynamically optimized mechanical ventilation regime for critically ill patients. Authors used a Markov decision process, including a reward system and a Q-learning approach, to find the optimized settings for positive end-expiratory pressure, fraction of inspired oxygen, and ideal body weight-adjusted tidal volume (Vt). They observed that VentAI would adjust settings more frequently when compared to human decisions, indicating a continuous reevaluation of the ventilation strategy to find the best fit for the individual patient.

**Figure 1.**
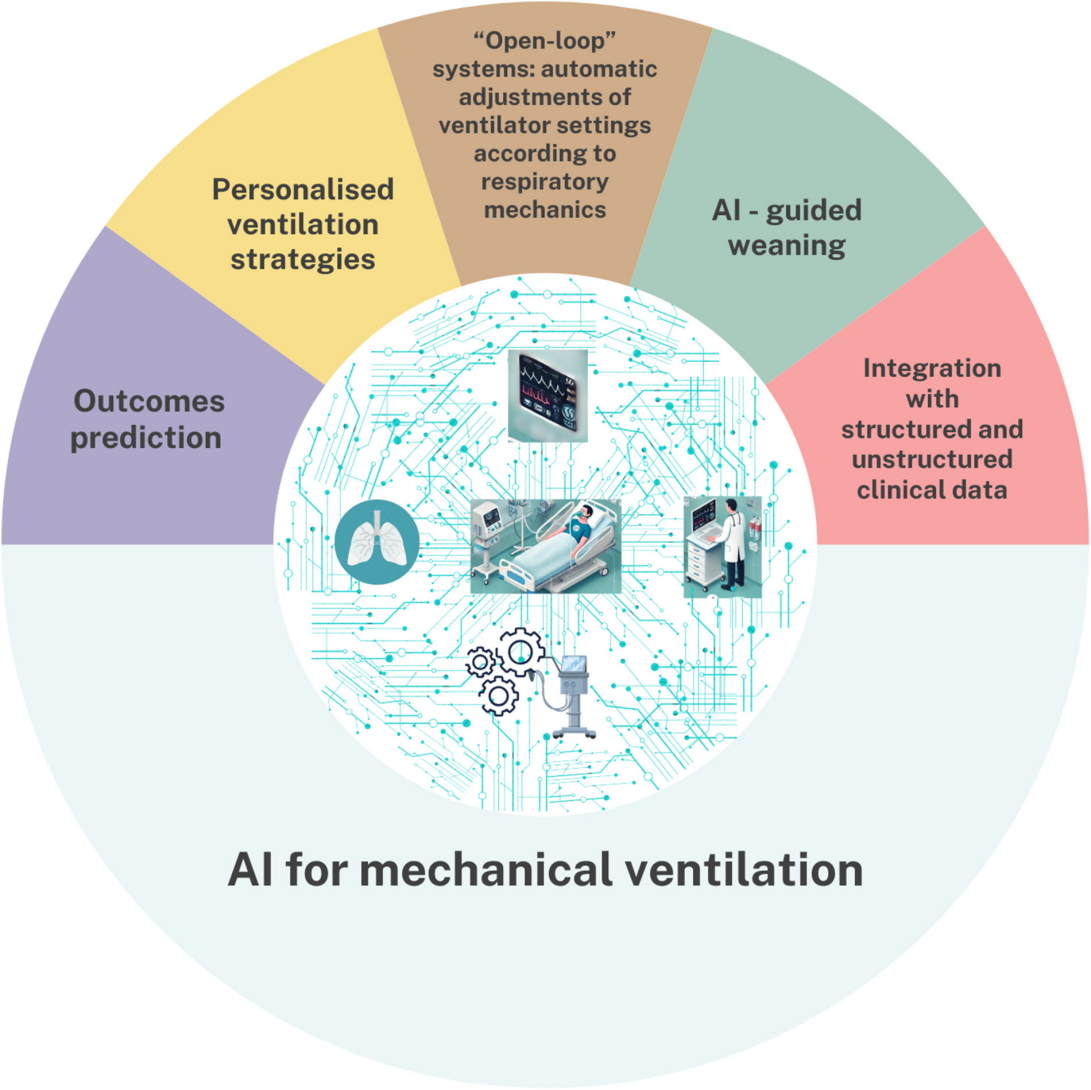
Infographic on artificial intelligence use for mechanical ventilation.

Identifying the right time for weaning initiation from mechanical ventilation is essential, given the associated risks and the lack of standardized protocols. An exponentially growing body of AI-related literature^2^ has been focused on the prediction of weaning timing and extubation success, demonstrating promising outcomes, including increased ventilator-free days and shorter ICU length of stay.^20^ These results highlight the potential of AI-guided weaning strategies and prediction models for helping clinicians in their decisions (Figure 1).

## Ethical and Practical Considerations

Despite its great potential, the application of AI for the management of mechanical ventilation is still at an early stage and requires a cautious and much less enthusiastic approach. The sensitive nature of health data necessitates robust measures to protect patient information, as well as consent of the patients with regard to data collection and use.^20^ Since most of the studies published to date are single center and retrospective in nature, prospective validation with clinical trials is needed to assess AI-guided ventilation. Data and codes should be open access, in order to improve reproducibility and transparency. In addition, the integration of AI into ventilation practices must be carefully managed to ensure that it enhances, rather than disrupts, the care process: physicians are responsible for their clinical practice and their decisions, and they should always bear in mind that bias is embedded in AI algorithms due to their “human” nature. As a consequence, accountability and responsibility for medical errors must be considered and shared to some extent with AI developers.

## Future Perspectives and Conclusions

The application of AI models in critical care is witnessing an unprecedented development. Research findings demonstrate that AI algorithms can reliably stratify subphenotypes of respiratory conditions, early predict which patients require mechanical ventilation and individualize ventilation strategies. With the integration of different data coming from mechanical ventilators, monitors, laboratories, imaging, and information from clinical documentation in real time, we might expect further improvements in future AI model performance. However, there are many challenges to overcome and results from present studies are not as encouraging as expected. Despite showing great potential for improving care for patients requiring mechanical ventilation, there are major limitations to consider and further research in this field is needed.
